# Steam vs. Hot Water Blanching Modulates Warmed-Over Flavor in Broccoli by Preserving Cellular Structure

**DOI:** 10.3390/foods15122216

**Published:** 2026-06-19

**Authors:** Mengrui Fan, Yuxiao Wang, Duanyin Gu, Junjie Gao, Hao Dong, Xin Sun, Qiyong Jiang, Rentang Zhang

**Affiliations:** 1 College of Food Science and Engineering, Shandong Agricultural University, Tai’an 271018, China; 2 Tai’an Academy of Agricultural Sciences, Tai’an 271000, China; 3 Shandong Meijia Group Co., Ltd., Rizhao 276800, China

**Keywords:** broccoli, blanching, warmed-over flavor, sulforaphane, volatile compounds

## Abstract

Blanching is a critical postharvest step that influences broccoli color, texture, flavor, and nutritional quality, and may affect the formation of warmed-over flavor (WOF) related volatile compounds under thermal processing. This study compares hot water blanching (HWB, 98 ± 1 °C, 30 to 150 s) and steam blanching (SB, 100 °C, 30 to 150 s) by analyzing color, texture, peroxidase activity, electronic nose, volatile compounds, sulforaphane content, and scanning electron microscopy (SEM) to determine how blanching conditions influence physicochemical attributes, cellular organization, and WOF-related volatile profiles in broccoli. Overall quality retention was superior with SB, particularly at 60 s, as evidenced by lower residual enzyme activity, improved firmness retention, better maintained cellular structure as observed microscopically, and higher sulforaphane retention relative to HWB. Multivariate analysis identified nine key volatile markers (variable importance in projection (VIP) > 1 and relative odor activity value (ROAV) > 1), including the WOF-associated aldehyde pentanal. Broccoli treated by SB for 60 s exhibited markedly lower levels of these aldehydes than samples subjected to HWB. Correlation analysis revealed a positive correlation between sulforaphane and sulfur-containing volatile compounds, including dimethyl disulfide and dimethyl trisulfide. This correlation mainly derived from the superimposed degradation of different precursor pools under thermal action: at high temperatures, dimethyl disulfide is one of the main volatile products generated from the thermal degradation of sulforaphane; meanwhile, during heating, intermediates derived from S-methyl-l-cysteine sulfoxide undergo thermal reactions to form dimethyl disulfide and dimethyl trisulfide. Collectively, these results support SB as an effective strategy to mitigate WOF while maintaining the nutritional quality of broccoli and potentially other cruciferous vegetables.

## 1. Introduction

Broccoli (*Brassica oleracea* L. var. *italica*) is packed with bioactive compounds like glucosinolates, sulforaphane, vitamin C, and phenolic compounds, and it is generally believed that the nutritional and health value of broccoli is significant [1,2]. Blanching is an industrially employed pretreatment in numerous processes that seek to stabilize colors by inactivating quality detracting enzymes (e.g., peroxidase, POD) [3], which subsequently extends the shelf life [4,5,6]. However, thermal treatments also introduce undesirable physicochemical changes, including the softening of tissues and loss of water-soluble nutrients. Additionally, these processes can promote the development of warmed-over flavor (WOF), which ultimately reduces consumer acceptance [7,8]. For minimally processed or ready-to-eat vegetable products, consumer rejection caused by WOF-related off-flavors may further reduce marketability, shorten the effective commercial shelf life, and lead to economic losses through product waste and decreased retail value [9,10].

Extensive research has examined the impact of blanching conditions on the quality of broccoli. A consistent finding across studies is that nutrient retention is strongly influenced by the blanching medium. In general, steam blanching (SB) and microwave blanching are more effective than hot water blanching (HWB) in preserving water-soluble and heat-sensitive nutrients, including vitamin C, total phenolics, and glucosinolates [2,11,12]. Quality losses are more pronounced in HWB. These losses are evident in the accelerated softening of firmness and the degradation of chlorophyll, both of which significantly affect the texture and color of the broccoli [2,13]. Recent studies have also explored the impact of processing on the functional properties of broccoli, including its antioxidant activity. These findings suggest that blanching, especially when followed by storage at low temperatures, can enhance the long-term stability and quality of broccoli [8]. Advancements in analytical tools and techniques such as instrumental and chemometric methods have made it easier to study the changes in flavor following processing. These methods facilitate profiling of taste and odor as well as identification of key volatile compounds, particularly sulfur compounds, which are commonly associated with thermal processes [7,14].

However, the production of lipid-oxidation aldehydes, which are key drivers of WOF, and their sensitivity to blanching medium and time have not been systematically explained [7]. Additionally, the mechanisms by which different heating methods (hot water blanching vs. steam blanching) influence cellular structure and subsequent volatile profile formation remain poorly understood. Therefore, an integrated framework linking microstructural changes, texture deterioration, and warmed-over flavor (WOF)-related volatile signals is needed to guide processing optimization.

Based on the underlying theory that different heating media affect tissue structure and oxidative reactions differently, we hypothesize that steam blanching, due to its rapid and uniform heating without direct water contact, will preserve cellular integrity more effectively than hot water blanching, thereby reducing the generation of WOF-related aldehydes and retaining higher levels of sulforaphane and overall quality. Therefore, this study aimed to investigate the differential effects of HWB and SB on the development of WOF in broccoli and to identify a processing method that effectively balances enzyme inactivation with flavor quality. The time-dependent inactivation of POD was used as an initial measure of blanching effectiveness. The analysis then focused on quantifying shifts in key WOF-related volatiles, including aldehydes (e.g., heptanal and hexanal) and sulfur-containing compounds (e.g., dimethyl disulfide). Nutritional quality was assessed by measuring sulforaphane retention and examining its correlation with sulfur volatiles resulting from glucosinolate hydrolysis. To establish a mechanistic understanding, microstructural data were integrated with macroscopic quality attributes such as texture and color as well as the volatile profiles through multivariate correlation and marker screening. This comprehensive approach provides insights for the development of targeted blanching strategies that minimize off-flavor formation while preserving desirable quality attributes in broccoli processing.

## 2. Materials and Methods

### 2.1. Materials

Fresh broccoli (*Brassica oleracea* L. var. *italica*, cv. You-xiu) was procured from a supermarket operated by Inzone Group Co., Ltd. (Taian, China). The selected heads were uniform in size, appearance, color, and maturity, and were free from mechanical damage and insect infestation. Catechol, hydrogen peroxide, and hydrochloric acid were purchased from Anpel Experimental Technology Co., Ltd. (Shanghai, China). Acetonitrile and ethyl acetate were of LC-MS grade from Macklin Biochemical Co., Ltd. (Shanghai, China). Sulforaphane reference standard (≥95.0%) and potassium phosphate buffer (0.1 mol/L, pH 6.5) were purchased from Yuanye Bio-Technology Co., Ltd. (Shanghai, China). Ultra-pure water was prepared using a Milli-Q water purification system (Millipore, Bedford, MA, USA). Unless otherwise noted, the chemicals and reagents used in this study were of analytical grade.

### 2.2. Blanching Treatments and Time Settings

Blanching conditions were adapted from a published method with minor modifications [15]. Two blanching treatments were applied at atmospheric pressure: HWB in boiling water and SB under saturated steam. For HWB, blanching was performed in boiling water maintained at 98 ± 1 °C using an electric hot pot (HGE2510, Midea Group Co., Ltd., Foshan, China). SB was performed with saturated steam at 100 °C. Individual broccoli florets, each weighing approximately 20 g, were placed in a single layer on the steamer, put in a covered stainless steel steamer (MZ-ZGC232366, Midea Group Co., Ltd., China), and continually blanched with steam. Samples were blanched for 0, 30, 60, 90, 120, and 150 s, respectively.

### 2.3. Color Measurement of Broccoli Treated with Different Blanching Methods

The color measurement of broccoli was conducted using an established method with slight modifications [16]. The color parameters (L*, a*, b*) of fresh and blanched broccoli were determined by CR 410 colorimeter (Minolta, Tokyo, Japan). The color change (ΔE) was calculated using Formula (1). In this formula, L*, a*, and b* represent the brightness, redness/greenness, and yellowness/blueness of the blanched product, respectively. Each group was repeated three times:

(1) ΔE = [(L* − L_0_*)^2^ + (a* − a_0_*)^2^ + (b* − b_0_*)^2^]^1/2^

where L*, a*, and b* are the color parameters of broccoli after blanching and L_0_*, a_0_*, and b_0_* are the color parameters of fresh broccoli.

### 2.4. Texture Profile Analysis of Broccoli Stems During Different Blanching Methods

The TPA of broccoli was conducted using an established method with slight modifications [15]. A texture analyzer (Bosin Tech, Shanghai, China) was used to conduct a compression test on broccoli stems to measure hardness, springiness, chewiness, and gumminess. For each test, three broccoli samples of similar size and shape were selected and a TA/100 compression plate probe was used. The instrument settings were as follows: pre-test speed of 2.00 mm/s, test speed of 1.00 mm/s, post-test speed of 5.00 mm/s, 2 s delay between compressions, trigger force of 3.0 g, and a fixed displacement distance of 3.0 mm.

### 2.5. Evaluation of the Chromogenic Method and Quantitative Determination of POD Activity in Broccoli

POD inactivation was evaluated using a guaiacol staining assay. In the presence of hydrogen peroxide, POD catalyzes the oxidation of guaiacol (o-methoxyphenol) to tetraguaiacol, which results in a visible brown color on the plant tissue [17]. To perform the assay, broccoli samples were collected after blanching and cooling at each time point, then cut longitudinally. A drop of 0.1% (*w*/*v*) guaiacol solution was applied to the cut surface, followed by a drop of 0.3% (*w*/*v*) hydrogen peroxide (H_2_O_2_) solution. The color change was visually observed and recorded [18]. If no color change occurred, the POD was considered largely inactivated. A brown color indicated that some POD activity remained.

Quantitative determination [19]: 2 g sample was mixed with 10 mL potassium phosphate buffer (0.1 mol/L, pH 6.5) for 1 min and centrifuged at 4000 r/min for 15 min to extract the crude enzyme solution. The POD activity was measured at 470 nm by spectrophotometer. The POD substrate solution was prepared by mixing 0.05 mL 99% guaiacol, 0.05 mL 30% H_2_O_2_, and 49.9 mL 0.1 mol/L potassium phosphate buffer (pH 6.5). The absorbance of the reaction mixture composed of 0.1 mL enzyme extract and 2.9 mL substrate solution was measured every 30 s. The activity of POD increased by 0.001 per minute at 470 nm as an enzyme activity unit (U), which was recorded as U/g FW. Enzyme activity was calculated according to Equation (2).

(2) POD activity (U/g FW) = (ΔA × V_t_)/(0.001 × V_s_ × Δt × FW)

In the formula: ΔA—change of absorbance; V_t_—total volume of enzyme extract (mL); V_s_—volume of the enzyme solution at the time of measurement (mL); Δt—time change (min); and FW—sample fresh weight (g).

### 2.6. Electronic Nose Analysis of Broccoli Odor Profiles

Odor profiles of broccoli samples were characterized using a PEN3 electronic nose (Airsense, Schwerin, Germany) equipped with ten metal-oxide sensors (Table S1) and controlled by Win Muster software (v1.6.2). Following a published procedure with minor modifications [20], broccoli samples from each blanching treatment were transferred to 40 mL headspace vials and sealed with Teflon–silicone septa. The vials were equilibrated at 25 °C for 60 min to allow for headspace stabilization. Subsequently, the probe was inserted into the vial headspace and sensor responses were recorded for 150 s at 5 s intervals. Between measurements, the sensor chamber was purged with clean carrier gas for 300 s to minimize carryover. The carrier gas flow rate and injection flow rate were both set to 400 mL/min.

### 2.7. Volatile Compounds Analyzed by HS-SPME–GC–MS

The relative contents of volatile compounds in the samples were analyzed using headspace solid-phase microextraction (HS-SPME) coupled with gas chromatography-mass spectrometry (GC-MS), (HP6890/5975C, Agilent, Santa Clara, CA, USA) following a published protocol with minor modifications [21]. Briefly, 2.0 g of sample was placed in a 20 mL headspace vial and sealed with a polytetrafluoroethylene–silicone septum. The vial was equilibrated at 50 °C for 20 min, and volatiles were subsequently adsorbed onto a 50/30 µm DVB/CAR/PDMS fiber (Supelco, Bellefonte, PA, USA) for 40 min. The fiber was then thermally desorbed in the GC injector at 250 °C for 2 min. Separation was performed on a DB-WAX capillary column with the following oven program: 40 °C for 3 min, increased to 120 °C at 5 °C/min and held for 2 min, then increased to 200 °C at 10 °C/min and held for 5 min. Helium was used as the carrier gas at 1.0 mL/min. The mass spectrometer was operated in electron ionization (EI) mode with an ion source temperature of 230 °C and a scan range of 35–500 *m*/*z*. Compounds were tentatively identified by matching mass spectra against the NIST14 library. Relative contents were expressed as the percentage of each peak area relative to the total peak area.

### 2.8. Analysis of Characteristic Flavor Compounds Based on Relative Odor Activity Value (ROAV)

The characteristic flavor components of broccoli with different blanching methods and time were determined by ROAV. When the ROAV of the compound is greater than or equal to 1, it can be judged as a typical flavor compound [22]. The calculation formula of ROAV value is as follows.

(3) ROAV = (C_A_/C_MAX_) × (T_MAX_/T_A_) × 100

In the formula: C_A_—relative content of volatile component, %; C_Max_—relative content of the maximum odor contribution component, %; T_A_—threshold of volatile components, μg/kg; T_Max_—maximum odor contribution component threshold, μg/kg.

### 2.9. Quantification of Sulforaphane by HPLC in Broccoli Treated with Different Blanching Methods

The sulforaphane content in broccoli was quantified using High-Performance Liquid Chromatography (HPLC, LC-20A, Shimadzu, Kyoto, Japan). A calibration curve was prepared from standard solutions (6.25–200 μg/mL), yielding a linear relationship: y = 1011.1x + 35,721 (R^2^ = 0.9907) (Figure S2). For sample preparation [23], 2.0 g of broccoli was hydrolyzed with hydrochloric acid at pH 3.0 at 25 °C for 0.5 h using the acid hydrolysis method, extracted with 20 mL ethyl acetate, and centrifuged; the supernatant was then evaporated to dryness at 45 °C. The residue was reconstituted in 5 mL acetonitrile and filtered through a 0.22 μm membrane prior to HPLC analysis. Chromatographic separation was performed on an AQ-C18 column (150 mm × 4.6 mm, 5 μm, Welch Materials, Shanghai, China) maintained at 30 °C. A gradient elution program was employed using deionized water (A) and acetonitrile (B) as the mobile phases. The initial condition was set at 80% A and 20% B. Over 20 min, the proportion of B was increased linearly to 60%, then to 80% at 25 min, and further to 100% at 28 min. The system returned to the initial ratio (80:20) at 35 min for column re-equilibration. The detection wavelength was 240 nm, with an injection volume of 10 μL and a flow rate of 1.0 mL/min. Sulforaphane content was calculated based on the peak areas and the calibration curve equation, and was expressed as a percentage on a dry weight basis (%, DW).

### 2.10. Scanning Electron Microscopy (SEM) Analysis of Broccoli Stems

Blanched broccoli stems were cut into blocks (1 × 1 cm) and freeze-dried. The dried samples were mounted on aluminum stubs with conductive carbon tape and sputter-coated with a thin layer of gold. Stem cross-sections were examined using a scanning electron microscope (Gemini 300, ZEISS, Oberkoc, Germany) operated under high vacuum at an accelerating voltage of 15 kV [24].

### 2.11. Statistical Analysis

All experiments were performed in triplicate, with results expressed as mean ± standard deviation. Differences among treatments were assessed by one-way ANOVA, followed by Duncan’s multiple range test for post hoc comparisons. Statistical significance was set at *p* < 0.05. Data processing and visualization were performed using SPSS Statistics (v27.0; IBM, Armonk, NY, USA), SIMCA (v14.1; Umetrics, Umeå, Sweden), OriginPro (v9.9; OriginLab, Northampton, MA, USA), GraphPad Prism (v10.0; GraphPad Software, San Diego, CA, USA), and TBtools-II (v2.331; Bio-Forge, Guangzhou, China).

## 3. Results and Discussion

### 3.1. Analysis of Blanching Methods on Broccoli Color Attributes

**Figure 1:**
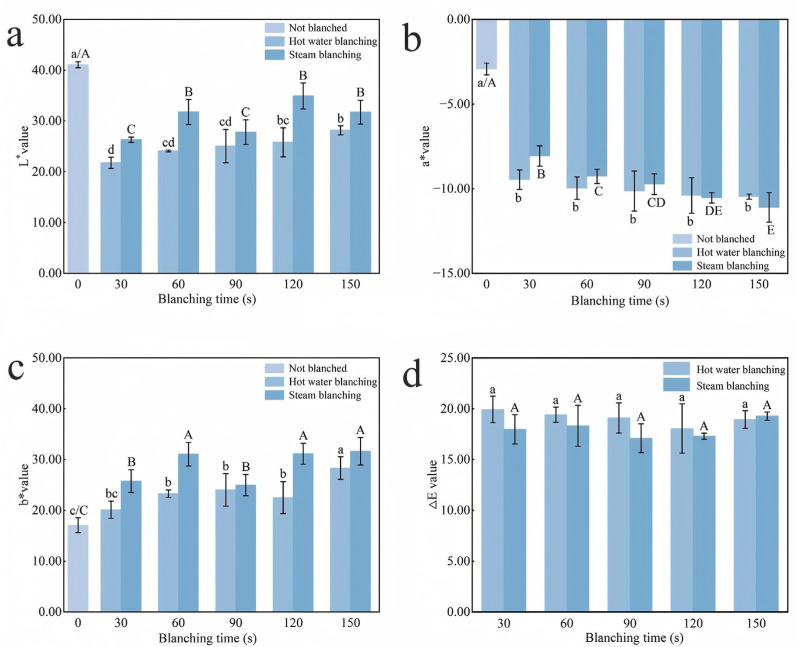
Color profiles of broccoli under different blanching methods. Results show (**a**) L* value, (**b**) a* value, (**c**) b* value, and (**d**) ΔE value. Means with different letters differ significantly at *p* < 0.05.

Figure 1 illustrates the CIELAB color coordinates (L*, a*, and b*) of broccoli after treatment with HWB and SB for varying times. Both blanching methods resulted in a decrease in L*, indicating a darkening of the tissue compared to the unblanched control. This decrease was more pronounced with HWB, likely due to a greater loss of water-soluble compounds, more extensive structural damage, and heat-induced reactions that together reduced lightness. Simultaneously, the a* value became more negative following heating, suggesting that the broccoli became greener. This increase in greenness may reflect thermal disruption of the cell membrane and matrix [25]. There are two ways that this phenomenon can be explained: first, breakdown of cell structure can lead to the release of chlorophyll, making it leak out of the cells into the extracellular space and increasing the green color [26]. A dramatic rise in greenness was previously noted after vacuum steam pulsed blanching of garlic scapes, which was explained by elevation in extracellular pigment concentration attributed to damage in the cell structure [24]. Conversely, the process of blanching removes the air in both surface trichomes and intercellular spaces. This changes the surface reflection properties of light, which can also be involved in improved green perception [5].

As the blanching time increased, the b* value tended to rise, indicating a shift toward a more yellow hue. The total color difference (ΔE) was calculated to capture the overall color deviation from fresh tissue. Both HWB and SB resulted in visible color changes (ΔE > 3.0), suggesting that blanching caused noticeable deviations from the unblanched sample. Notably, the SB values were consistently lower than those of HWB, indicating that SB better preserves the original color of broccoli over a longer period compared to HWB [11].

### 3.2. TPA Analysis of Broccoli Under Different Blanching Methods

**Figure 2:**
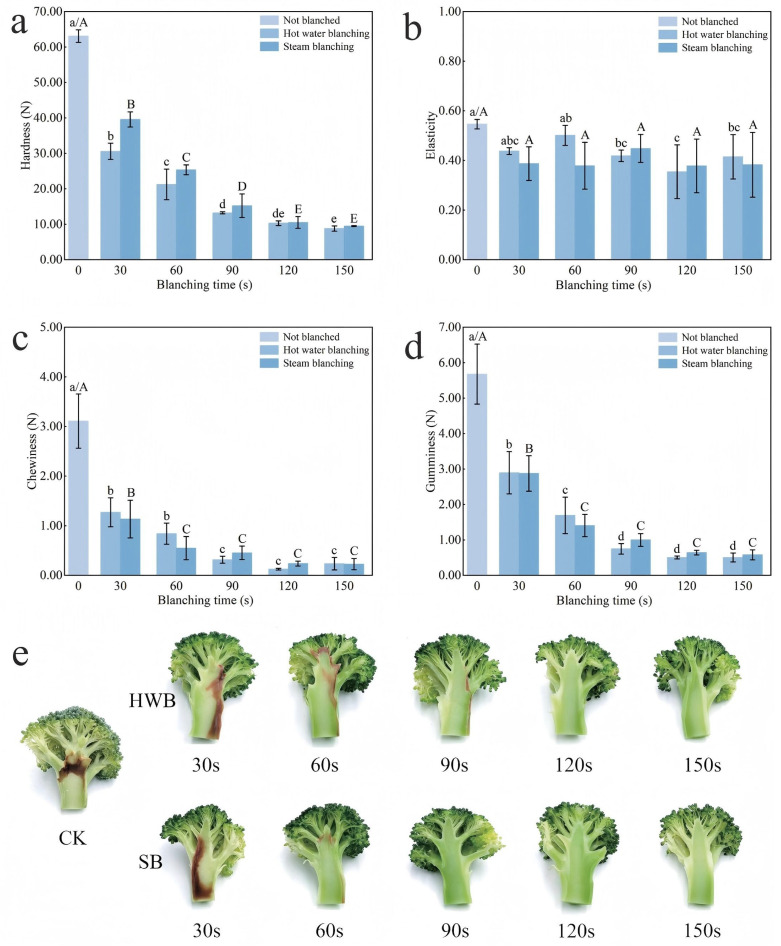
Texture profile and POD inactivation of broccoli under different blanching methods. Results show (**a**) hardness, (**b**) springiness, (**c**) chewiness, and (**d**) gumminess. Means with different letters differ significantly at *p* < 0.05. (**e**) Visual assessment of peroxidase activity, (CK) unblanched control, (HWB) hot water blanching for 30 to 150 s, and (SB) steam blanching for 30 to 150 s.

One important sensory property is texture which has a great impact on consumer acceptance [25]. Figure 2a,c,d show that hardness, chewiness, and gumminess decreased significantly with increased blanching time for both blanching methods. This suggests that the broccoli tissues progressively softened during thermal processing. This softening is likely associated with heat-induced breakdown of the cell wall and middle lamella structures. Specifically, high temperatures may accelerate the solubilization and depolymerization of pectin, weakening the bonds between cells. Meanwhile, the disorientation of cellulose microfibrils and membrane rupture reduce cell turgor, ultimately leading to a loss of firmness and chewiness [27,28].

**Figure 6:**
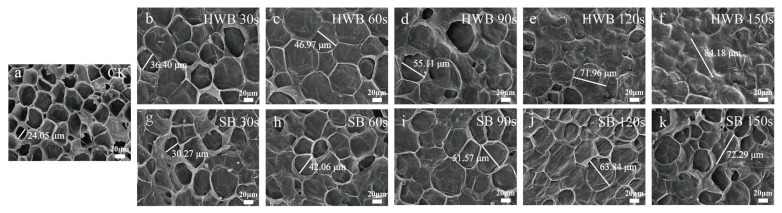
SEM images of broccoli subjected to different blanching methods. The results show that as blanching time is prolonged, the degree of collapse in broccoli cells becomes more severe, the cavities are enlarged, and the collapse under HWB is more serious than that under SB. (**a**) CK; (**b**) HWB 30 s; (**c**) HWB 60 s; (**d**) HWB 90 s; (**e**) HWB120 s; (**f**) HWB 150 s; (**g**) SB 30 s; (**h**) SB 60 s; (**i**) SB 90 s; (**j**) SB 120 s; (**k**) SB 150 s.

HWB consistently resulted in softer tissues compared to SB, as evidenced by lower hardness values at comparable blanching intervals. For instance, after 30 s of treatment, the hardness decreased to 30.58 N with HWB, while with SB the hardness remained higher at 39.57 N. This difference is likely due to the immersion medium in HWB, which promotes solute leaching and pectin loss, leading to faster softening. Additionally, high temperatures may accelerate the degradation of pectins through β-elimination reactions, further weakening the middle lamella and contributing to the softening process [29]. These findings indicate that the rate and degree of cell wall decay during the blanching process are regulated by the heat transfer medium (water or steam) [28]. Research has indicated that blanching changes the cell wall structure, which is one of the major factors in softening of the texture [21]. Later microscopic structural analysis also proved that HWB induced more drastic collapse in the cell wall structure as compared to SB. Notably, after prolonged SB, the texture changed dramatically. The hardness of broccoli decreased sharply from approximately 62 N in fresh samples to about 10 N after 150 s of SB, representing a residual firmness of 16% relative to the fresh samples. Studies have shown that broccoli with medium firmness (20–40% of raw firmness) receives the highest consumer liking, while low-firmness samples (5–20% of fresh samples) are still considered acceptable. Therefore, the residual hardness of 10 N after 150 s SB falls within the marginally acceptable texture range for consumers [30]. This marked softening is closely linked to the cellular collapse observed by SEM (Figure 6), as thermal degradation of pectin leads to cell separation and loss of tissue integrity, which in turn corresponds to the sensory perception of increased tenderness and slight mushiness after prolonged heating. Elasticity generally declined with the rise in blanching period (Figure 2b).

### 3.3. POD Inactivation During Different Blanching Methods

Figure 2e shows that the freshly cut surface of unblanched broccoli rapidly developed a reddish-brown color after the sequential application of guaiacol and H_2_O_2_, indicating high POD activity in the control. This qualitative staining response follows the principle of the guaiacol-H_2_O_2_ assay, where POD catalyzes the oxidation of guaiacol, producing colored products that lead to visible browning. In addition to serving as an indicator of activity, the oxidative reactions mediated by POD may also contribute to quality deterioration by facilitating the interaction of reactive intermediates with H_2_O_2_. This reaction promotes the oxidation of weak substrates, modifies pigments, and accelerates lipid peroxidation [3].

The guaiacol staining reaction progressively decreased as the blanching time increased. Notably, the cut surfaces did not turn brown after 120 s of HWB or 90 s of SB, indicating that POD activity was nearly completely inactivated in these cases. This qualitative observation was consistent with the results of the quantitative POD assay (Figure S1). POD activity significantly decreased in SB over an equivalent treatment period. For example, 60 s of SB reduced POD activity from 2660.03 to 595.47 U/g FW, demonstrating inactivation of the enzyme. These denaturation effects are typically attributed to heat, with prolonged heating causing the loss of the enzyme’s native structure and a subsequent reduction in its catalytic activity in the guaiacol-H_2_O_2_ reaction [31,32]. Decreases in POD activity over time due to thermal processing have been extensively documented [18,19], and accelerated POD reduction with increasing SB times is in line with previous results [4]. In summary, the guaiacol staining reaction showed that SB inactivated POD more quickly than HWB when both treatments were applied for the same time. This advantage can be attributed to the higher heat transfer efficiency of SB, as the condensing steam on the product surface rapidly provides latent heat, leading to faster achievement of enzyme-inactivating temperatures. These method-dependent differences align with previous studies which have demonstrated that enzyme inactivation kinetics are strongly influenced by the nature of the blanching medium, with SB generally exhibiting higher inactivation efficiency than HWB [18].

### 3.4. Analysis of Broccoli Flavor Patterns During Blanching Using an E-Nose

**Figure 3:**
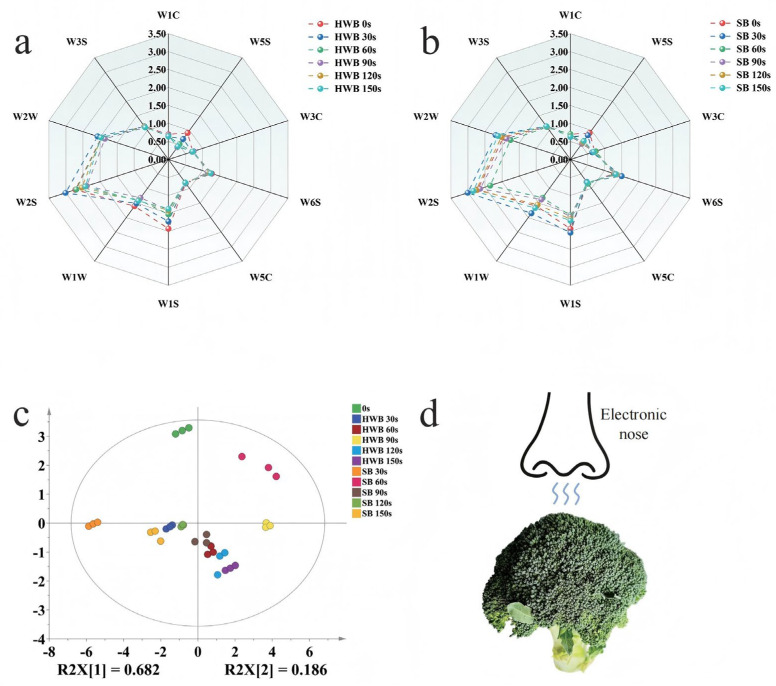
E-nose odor fingerprints of broccoli during blanching. (**a**) Radar chart of the responses from ten electronic nose (E-nose) sensors for hot-water blanching. (**b**) Radar chart of the responses from ten E-nose sensors for steam blanching. (**c**) Principal component analysis (PCA) results for flavor of broccoli. (**d**) Schematic illustration of electronic nose detection for characterizing the overall odor profile of broccoli samples.

PCA is an unsupervised pattern recognition method that reduces data dimensionality by extracting the most significant information as principal components. As shown in Figure 3c, the first two principal components accounted for 86.8% of the total variance. This indicates that the PCA model successfully captured the main odor variations among broccoli samples during blanching. This approach is commonly used to interpret E-nose data in food flavor studies [7]. The unblanched control was grouped in the negative region of PC1, clearly separating it from all blanched treatments. This suggests that blanching caused a significant shift in the overall headspace odor profile. Similar heat-driven changes in volatile patterns have also been observed in other vegetables [20,21].

The sensor response patterns further supported these PCA observations. At matched time points, steam-blanched samples showed relatively higher signals on W1W (sulfur-responsive) and W2W (aromatic/organic-responsive) than samples blanched with hot water (Figure 3a,b), implying better retention of sulfur-related odor notes under steam conditions. Given that sulfur-containing volatiles are central contributors to the characteristic aroma of cruciferous vegetables, these E-nose responses collectively indicate that SB better preserves broccoli’s typical odor attributes compared to HWB [33].

### 3.5. Differences in Volatile Profiles Analyzed by Multivariate Data Analysis

**Figure 4:**
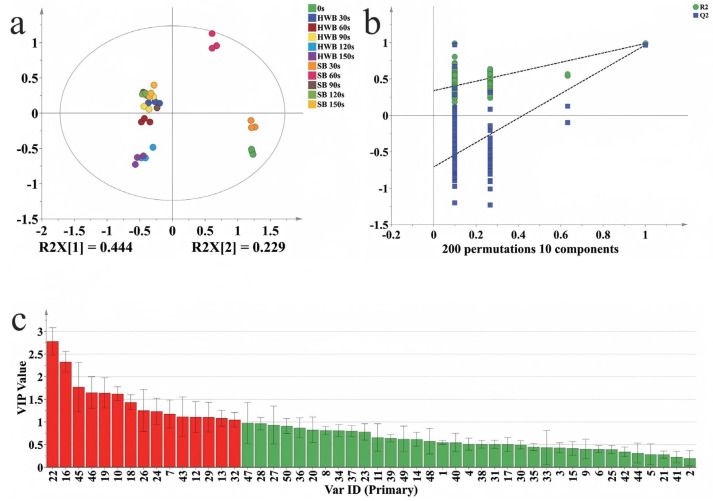
Supervised multivariate discrimination of broccoli volatiles across blanching treatments. (**a**) Score plot of the orthogonal partial least squares discriminant analysis (OPLS-DA) model. (**b**) Validation of the OPLS-DA model. (**c**) Variable importance in the projection (VIP) diagram (red bars indicate compounds with VIP > 1 (principal markers), green bars indicate compounds with VIP < 1), corresponding to the following compounds: 22 pentanal, 16 1-pentanol, 45 dimethyl disulfide, 46 dimethyl trisulfide, 19 2-ethyl-1-ethanol, 10 pentadecane, 18 1-octen-3-ol, 26 heptanal, 24 hexanal, 7 2,6,10-trimethyltridecane, 43 2-methylbutyl isothiocyanate, 12 1-hexanol, 29 benzaldehyde, 13 Z-3-ethylene-1-ol, and 32 decanal; the full list of compound names for all IDs numbers is provided in Supplementary Table S4. One-way ANOVA was used for statistical analysis. Detailed statistical results, including VIP values, *p* values, and mean ± SD with significance annotations, are provided in Supplementary Table S4.

To quantify treatment-driven separation in volatile composition, an OPLS-DA model was constructed (Figure 4a). The model showed good performance (R2X = 0.973, R2Y = 0.94, Q2 = 0.852) and permutation testing supported model validity (Q2 intercept = −0.69; Figure 4b). In the score plot, SB at 30–60 s was generally positioned on the right side of the first component, whereas HWB samples across time and longer SB treatments (90 to 150 s) were mainly on the left, indicating a combined effect of blanching medium and time rather than a purely “method-only” separation. Replicates from HWB were more tightly clustered than those from SB, while SB showed greater dispersion across time, suggesting stronger time sensitivity of the volatile profile under steam conditions.

**Figure 5:**
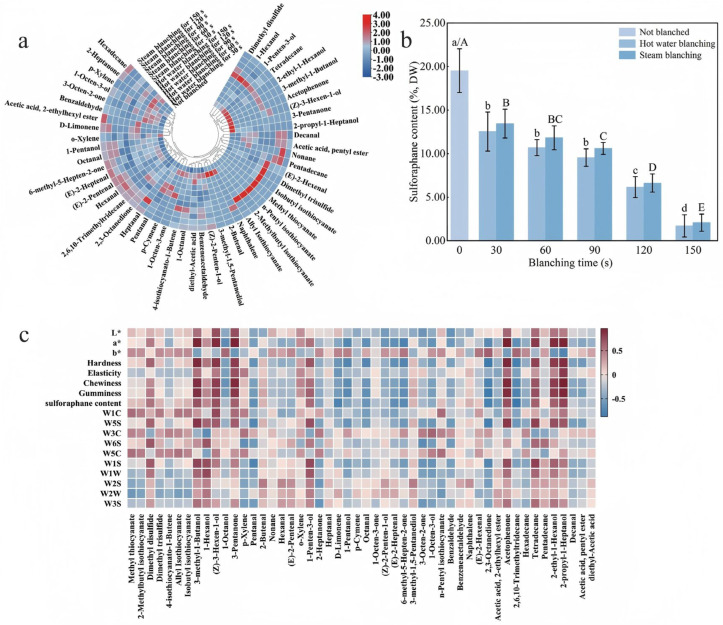
Integrated changes in broccoli volatiles, sulforaphane, and quality relationships during blanching. (**a**) Heatmap showing changes in the relative content of volatile compounds across different blanching treatments; color intensity represents the relative abundance of each volatile compound. (**b**) Sulforaphane content as a function of blanching time (different letters indicate significant differences among treatments (*p* < 0.05)). (**c**) Correlation heatmap showing the relationships among color attributes, texture traits, sulforaphane content, E-nose sensor responses, and volatile compounds. Red indicates positive correlations, whereas blue indicates negative correlations.

The contribution of each variable was quantified by calculating VIP scores from the OPLS-DA model. VIP screening identified 15 discriminant volatiles (VIP > 1; Figure 4c). Additionally, statistical significance among groups was further evaluated by one-way ANOVA (Supplementary Table S4). All volatile compounds showed highly significant differences among groups (*p* < 0.001), indicating a strong impact of processing method on volatile composition. Nine of these were simultaneously classified as key odor-active contributors (ROAV > 1) (Table S3) and discriminant markers (VIP > 1), namely, valeraldehyde, 1-pentanol, dimethyl disulfide, dimethyl trisulfide, 1-octen-3-ol, heptanal, hexanal, 1-hexanol, and decanal. Combined with the analysis in Figure 5a and Table S2, these markers are consistent with two primary routes: accumulation of lipid oxidation-derived aldehydes, which has been reported during blanching of other foods such as broad beans and mushrooms [6,22,34], and thermal conversion of endogenous sulfur-containing precursors (e.g., glucosinolates) [35]. In HWB, pentanal—associated with grassy/fatty off-notes typical of WOF—increased markedly with treatment time, reaching 31.78% in HWB-150 s. This change may be related to the accelerated cell damage, lipid exposure, and potential oxidation trend in the direct water environment [10]. Similarly, hexanal and heptanal, which were also identified as lipid oxidation-derived aldehydes, exhibited different accumulation behaviors under HWB conditions. Hexanal showed an early increase followed by a gradual decline, whereas heptanal was detected only at the early stage of treatment and subsequently not detected, suggesting differences in volatility and reactivity among aldehyde species derived from lipid oxidation cascades. In contrast, heptanal and hexanal were not detected in SB-60 s, suggesting that moderate steaming may reduce the accumulation of WOF-associated aldehydes under the tested conditions [10]. This phenomenon may be related to the preservation of cellular structural integrity during steam treatment. It has been reported that steam blanching better maintains the structural integrity of plant tissues compared with hot water blanching, and also avoids excessive swelling and disintegration of the cell walls [36]. Meanwhile, thermal processing has been shown to accelerate the thermal degradation of unsaturated fatty acids, thereby promoting the generation of aliphatic aldehydes that contribute to off-flavors [37]. Consequently, the absence of these lipid oxidation-related aldehydes in the SB-60 s sample likely reflects the protective effect of moderate steam treatment on cell membrane integrity, which limits the exposure of membrane lipids to oxidative environments and suppresses the initiation of the lipid oxidation cascade. Notably, when the steam blanching time was prolonged beyond 60 s, the relative contents of these aldehydes increased (Table S2). This observation further supports the proposed mechanism: prolonged thermal exposure gradually compromises membrane integrity, allowing lipid oxidation to resume and off-flavor aldehydes to accumulate. For sulfur volatiles, dimethyl disulfide and dimethyl trisulfide peaked at SB for 60 s (7.21% and 11.22%), then decreased with longer steaming. This transient response likely indicates that moderate steaming promotes the heat-driven conversion of glucosinolate-related precursors. As a result, sulfur volatiles are formed preferentially over enzymatic products [35]. The subsequent decrease with extended SB suggests progressive thermal loss/degradation under stronger heating, consistent with reports in other cruciferous vegetables (e.g., kale) showing accelerated loss of flavor-related compounds at higher heat intensity [32]. In contrast, HWB resulted in broad losses of sulfur-containing compounds, including isothiocyanates, likely due to water-phase extraction, which can weaken characteristic broccoli notes; this aligns with prior reports that thermal processing reduces sulfur volatiles in broccoli and that HWB is associated with greater flavor loss [7,14]. It should be noted that volatile components were analyzed using relative abundance (peak area percentage) in this study. Although this approach is widely applied for comparative analysis of volatile profiles, it has inherent limitations. HWB and SB induce different degrees of cellular damage in broccoli tissues, which affects the total peak area of volatiles. Accordingly, relative content cannot fully represent the absolute concentration of each compound. Semi-quantitative analysis with an internal standard would effectively improve methodological rigor, and this optimized approach will be adopted in future studies.

### 3.6. Dynamic Changes in Sulforaphane During Different Blanching Methods

As shown in Figure 5b, with increasing blanching time, the sulforaphane content significantly decreased in both the HWB and SB groups, with a more pronounced reduction observed in the HWB group. This phenomenon may be related to heat-induced degradation of sulforaphane and its precursors [38]. At the same time, as myrosinase is a key enzyme in the conversion of glucosinolates, its thermal stability is also widely considered to be an important factor affecting the content of active substances in cruciferous vegetables [17]. Blanching treatments can simultaneously promote degradation of sulforaphane and its precursors, thereby reducing the measurable sulforaphane level, particularly under more severe heating conditions [39]. A similar blanching-associated decrease in sulforaphane has also been reported previously [12,23].

Sulforaphane retention was also associated with the volatile profile described in Section 3.5. It is important to note that relatively high levels of sulforaphane were also maintained in the SB-60 s treatment, and a number of desirable volatiles such as allyl isothiocyanate were retained as well (Table S2). This co-retention tendency indicates that the water-soluble concentration of compounds might be minimized due to the presence of SB while offering sufficient heat to favor the production or maintenance of certain flavor compounds; as a result, this helps to maintain a more balanced broccoli aroma profile [23]. In contrast, HWB caused a greater loss of sulforaphane and coincided with a more unfavorable volatile pattern. This was characterized by a significant increase in pentanal (associated with WOF), a trend that aligned with the volatile substance changes under long-term hot water treatment [10]. Overall, changes in sulforaphane levels not only reflect the nutritional quality of broccoli but also serve as a key indicator of the impact of blanching methods on the overall flavor precursor pool of broccoli [1,33].

### 3.7. Correlation Heatmap Linking Quality Attributes, Sulforaphane, and Flavor-Related Markers in Blanched Broccoli

Figure 5c presents the correlation heatmap linking color attributes, texture traits, sulforaphane content, E-nose signals, and individual volatiles across blanching treatments. Sulforaphane was positively associated with several sulfur-containing volatiles, most notably dimethyl disulfide and dimethyl trisulfide. Studies have shown that sulforaphane undergoes thermal degradation in aqueous solution at 100 °C, generating dimethyl disulfide as one of its volatile decomposition products [40]. Meanwhile, S-methyl methanethiosulfinate, an organosulfur compound released from S-methyl-l-cysteine sulfoxide in Brassica vegetables, thermally decomposes at 80–200 °C to yield dimethyl disulfide and dimethyl trisulfide as major products [41]. Furthermore, glucosinolates and S-methyl-l-cysteine sulfoxide coexist as the primary sulfur-containing secondary metabolites in Brassica vegetables, and their levels vary synchronously with variety and cultivation conditions (e.g., season, temperature, and sulfur supply) [42]. Therefore, the observed positive correlation likely reflects the superposition of two independent thermal degradation processes: the thermal decomposition of sulforaphane itself promotes the formation of dimethyl disulfide, while the thermal degradation of S-methyl methanethiosulfinate simultaneously produces both dimethyl disulfide and dimethyl trisulfide. Because sulforaphane declines with heating [38], these correlations reflect between-sample differences across treatments, not synchronous time-course changes. Furthermore, color attributes were significantly associated with specific volatile compounds. For example, a* value (greenness) was negatively correlated with (E)-2-heptenal and other aldehydes related to WOF. This pattern is consistent with typical thermal effects on volatile profiles during heat treatment, which are often accompanied by the formation of lipid-derived aldehydes associated with green and fatty notes [10]. In addition, heat treatment facilitates the release and redistribution of chlorophyll, which in turn increases the greenness value [26].

E-nose–compound relationships further supported the GC–MS patterns. Sensors W1W and W2W had a positive correlation with sulfur volatiles such as dimethyl disulfide, implying that they are sensitive to the typical notes of sulfur in cruciferous vegetables [33]. The pentyl acetate ester, which is a primary contributor to fruity aromas, awas positively correlated with sensor W5C. However, there was a broad negative correlation between W1S and various volatiles, indicating that W1S is likely to signal an overall change in the headspace profile and not an individual compound category. Combining the above results, they suggest that the E-nose can record treatment-specific differences in the important clusters of volatiles, and can be used to supplement the marker analysis via GC-MS.

### 3.8. Analysis of Broccoli Microstructure During Blanching Methods

As revealed in Figure 6, the initial sample had a full cellular structure characterized by discrete cell walls and sharp limits. With longer blanching times there was a higher degree of cellular breakdown, which could be because of degradation of pectin and development of intercellular fissures due to high temperatures [43]. This observation is in line with the heat-induced modifications in the structure of pectin and the softening processes of tissues, since the pectin degradation caused by heat alters the integrity of the cell wall, which in turn loosens associations between cells, causing structural breakdown [27].

HWB resulted in more structural damage than SB with the same treatment time. This observation is similar to those in the literature indicating that the blanching medium can have a significant influence on the magnitude of microscopic damage [11]. In HWB, direct exposure to hot water causes thermal injury and promotes the loss of intracellular components. Excessive water temperatures destroy the cell membrane, leading to cellular damage. This contributes to rupture of the microstructure, resulting in higher levels of structural damage in the HWB samples [26]. In comparison, SB is mostly used to provide heat by condensing steam, and has minimum leaching rates that ensure possible preservation of cellular integrity. Such variations in medium-dependent microstructural preservation have also been noted in other vegetable samples that were blanched with steam versus water [43].

## 4. Conclusions

There are significant differences in enzyme inactivation, texture preservation, flavor retention, and microstructural integrity due to variations in heat transfer and exchange processes among different blanching methods. Overall, SB for 60 s demonstrated clear benefits in terms of maintaining quality. Compared to HWB, SB significantly reduced enzyme activity, better preserved sample hardness and cell structure, and was more effective in retaining sulforaphane. In this study, GC-MS, HPLC, electronic nose technology, and multivariate statistical analysis were employed to examine how volatile flavor compounds in broccoli changed with various blanching methods and time. Notably, nine volatile compounds—valeraldehyde, 1-pentanol, dimethyl disulfide, dimethyl trisulfide, 1-octen-3-ol, heptanal, hexanal, 1-hexanol, and decanal—were identified as key volatile flavor compounds (ROAV > 1) and differential markers (VIP > 1). Correlation analysis revealed that sulforaphane was positively correlated with sulfur-containing volatiles such as dimethyl disulfide, likely due to the superposition of two independent thermal degradation processes: the thermal decomposition of sulforaphane itself promotes the formation of dimethyl disulfide, while the thermal degradation of S-methyl methanethiosulfinate simultaneously produces both dimethyl disulfide and dimethyl trisulfide. These observations indicate a relationship between cellular structural disruption and the formation of undesirable volatile compounds. This research provides a theoretical foundation for quality control in broccoli processing, with the aim of minimizing WOF formation while preserving characteristic flavors. However, this study has certain limitations. Only fixed blanching time and temperature combinations were investigated, which may not fully capture the dynamic changes in volatile compounds under different processing conditions. In addition, only a limited number of broccoli cultivars were examined. Future studies could explore the response of different broccoli varieties to blanching, integrate metabolomics and transcriptomics to investigate flavor precursor conversion, and extend this approach to other cruciferous vegetables for a more universal hot processing strategy.

## Data Availability

The original contributions presented in the study are included in the article/Supplementary Material, further inquiries can be directed to the corresponding author.

## Supplementary Materials

The following are available online at https://www.mdpi.com/article/10.3390/foods15122216/s1, Table S1: Ten sensors and their main applications of E-nose; Table S2: The composition and relative content of volatile flavor compounds in broccoli under different blanching methods and time; Table S3: Relative odor activity values (ROAV ≥ 1) of broccoli volatile flavor compounds detected under different blanching methods and time; Table S4: VIP values, ANOVA significance and relative contents of volatile compounds in broccoli under different blanching treatments; Figure S1: Peroxidase activity of broccoli under different blanching times; Figure S2: The standard curve of sulforaphane by HPLC analysis.
