# Supplementary material for: Steam vs. Hot Water Blanching Modulates Warmed-Over Flavor in Broccoli by Preserving Cellular Structure
Source: Foods. 2026 Jun 19;15(12):2216. doi: 10.3390/foods15122216 (PMC13298270; doi:10.3390/foods15122216)
Supplement: Supplementary file 1 [file foods-15-02216-s001.zip › foods-4363518-supplementary.pdf]

Supporting Information

Steam vs. Hot Water Blanching Modulates Warmed-Over Flavor in Broccoli by Preserving Cellular Structure

Mengrui Fan [1,†], Yuxiao Wang [1,†], Duanyin Gu [2], Junjie Gao [2], Hao Dong [3], Xin Sun [1], Qiyong Jiang [1] and Rentang Zhang [1,*]

[1] College of Food Science and Engineering, Shandong Agricultural University, Tai'an 271018, China
[2] Tai'an Academy of Agricultural Sciences, Tai'an 271000, China
[3] Shandong Meijia Group Co., Ltd., Rizhao 276800, China
* Correspondence: rentangzhang@163.com
† These authors contributed equally to this work.

Table S1. Ten sensors and their main applications of E-nose.

Array serial number	Sensor name	Main applications
1	W1C	Sensitive to aromatic compounds
2	W5S	Broad sensitivity and very sensitive to nitrogen oxides
3	W3C	sensitive to ammonia and aromatic compounds
4	W6S	mainly sensitive to hydrogen
5	W5C	sensitive to alkenes and aromatic compounds
6	W1S	sensitive to methane
7	W1W	sensitive to sulfides compounds
8	W2S	sensitive to alcohols, partially aromatic compounds
9	W2W	sensitive to aromatic compounds and organic sulfides
10	W3S	mainly sensitive to alkenes

Table S2. The composition and relative content of volatile flavor compounds in broccoli under different blanching methods and time

Serial Number	Name	Relative content (Mean ± SD)/%										
		Un-blanched	Hot water blanching for 30 s	Hot water blanching for 60 s	Hot water blanching for 90 s	Hot water blanching for 120 s	Hot water blanching for 150 s	Steam blanching for 30 s	Steam blanching for 60 s	Steam blanching for 90 s	Steam blanching for 120 s	Steam blanching for 150 s
	Hydrocarbon											
	Total content	1.28	1.13	1.83	1.69	2.46	3.47	3.42	1.30	6.18	7.61	2.96
1	P-xylene	0.16±0.02^{c}	0.00±0.00^{d}	0.21±0.02^{b}	0.31±0.02^{a}	0.00±00^{d}	0.16±0.05^{c}	0.00±0.00^{d}	0.03±0.01^{d}	0.00±0.00^{d}	0.00±0.00^{d}	0.00±0.00^{d}
2	Nonane	0.00±0.00^{c}	0.00±0.00^{c}	0.00±0.00^{c}	0.03±0.01^{b}	0.00±0.00^{c}	0.00±0.00^{c}	0.00±0.00^{c}	0.00±0.00^{c}	0.00±0.00^{c}	0.05±0.04^{a}	0.00±0.00^{c}
3	O-xylene	0.30±0.15^{a}	0.12±0.04^{cde}	0.22±0.05^{abc}	0.00±0.00^{f}	0.22±0.07^{ab}	0.21±0.11^{abcd}	0.00±0.00^{f}	0.11±0.03^{de}	0.00±0.00^{f}	0.09±0.01^{ef}	0.14±0.02^{bcde}
4	D-limonene	0.00±0.00^{c}	0.00±0.00^{c}	0.00±0.00^{c}	0.00±0.00^{c}	0.22±0.07^{a}	0.16±0.01^{b}	0.00±0.00^{c}	0.00±0.00^{c}	0.00±0.00^{c}	0.21±0.05^{ab}	0.17±0.01^{b}
5	P-cymene	0.00±0.00^{b}	0.07±0.04^{a}	0.00±0.00^{b}	0.00±0.00^{b}	0.09±0.06^{a}	0.06±0.02^{a}	0.00±0.00^{b}	0.00±0.00^{b}	0.00±0.00^{b}	0.00±0.00^{b}	0.00±0.00^{b}
6	Naphthalene	0.00±0.00^{c}	0.12±0.02^{a}	0.00±0.00^{c}	0.00±0.00^{c}	0.00±0.00^{c}	0.00±0.00^{c}	0.00±0.00^{c}	0.00±0.00^{c}	0.10±0.03b	0.00±0.00^{c}	0.00±0.00^{c}
7	2,6,10-trimethyl tridecane	0.00±0.00^{d}	0.31±0.12^{cd}	0.69±0.10^{c}	0.71±0.06^{c}	1.59±0.35^{b}	2.28±0.50^{a}	0.44±0.05^{cd}	0.48±0.17^{cd}	1.72±0.72^{ab}	2.03±0.20^{ab}	1.81±0.14^{ab}
8	Hexadecane	0.00±0.00^{d}	0.00±0.00^{d}	0.51±0.02^{a}	0.42±0.09^{b}	0.00±0.00^{d}	0.35±0.03^{c}	0.00±0.00^{d}	0.32±0.01^{c}	0.00±0.00^{d}	0.00±0.00^{d}	0.53±0.01^{a}
9	Tetradecane	0.82±0.21^{a}	0.20±0.12^{c}	0.20±0.01^{c}	0.22±0.09^{c}	0.34±0.02^{bc}	0.25±0.02^{c}	0.73±0.16^{a}	0.36±0.03^{bc}	0.48±0.13^{b}	0.33±0.03^{bc}	0.31±0.01^{bc}
10	Pentadecane	0.00±0.00^{d}	0.31±0.05^{d}	0.00±0.00^{d}	0.00±0.00^{d}	0.00±0.00^{d}	0.00±0.00^{d}	2.25±0.37^{c}	0.00±0.00^{d}	3.88±0.28^{b}	4.90±0.64^{a}	0.00±0.00^{d}
	Alcohols											

	Total content	17.29	24.56	21.77	27.69	18.35	13.82	13.65	24.57	25.34	27.08	26.82
11	3-methyl-1-butanol	1.73±0.57^{a}	0.00±0.00^{c}	0.00±0.00^{c}	0.00±0.00^{c}	0.00±0.00^{c}	0.00±0.00^{c}	1.07±0.18^{b}	0.00±0.00^{c}	0.00±0.00^{c}	0.00±0.00^{c}	0.00±0.00^{c}
12	1-hexanol	1.27±1.11^{b}	0.33±0.05^{c}	0.38±0.03^{c}	0.93±0.19^{bc}	0.23±0.03^{c}	0.26±0.04^{c}	3.85±0.28^{a}	0.55±0.10^{c}	0.27±0.02^{c}	0.32±0.03^{c}	0.72±0.38^{bc}
13	(Z)-3-hexen-1-ol	3.33±0.39^{a}	0.00±0.00^{d}	0.00±0.00^{d}	0.00±0.00^{d}	0.00±0.00^{d}	0.00±0.00^{d}	0.74±0.14^{c}	1.90±0.29^{b}	0.00±0.00^{d}	0.00±0.00^{d}	0.00±0.00^{d}
14	1-octanol	0.00±0.00^{f}	1.10±0.23^{b}	0.87±0.15^{bcd}	0.83±0.19^{cd}	0.67±0.04^{de}	0.56±0.06^{e}	0.74±0.14^{de}	1.41±0.26^{a}	1.05±0.04^{bc}	0.90±0.02^{bcd}	1.06±0.09^{bc}
15	1-penten-3-ol	0.47±0.26^{b}	0.37±0.06^{bcd}	0.27±0.05^{bcde}	0.4±0.08^{bc}	0.17±0.07^{de}	0.22±0.06^{cde}	0.70±0.15^{a}	0.31±0.14^{bcde}	0.15±0.03^{f}	0.23±0.04^{cde}	0.29±0.03^{bcde}
16	1-pentanol	0.36±0.12^{f}	16.02±1.47^{bc}	14.95±2.03^{cd}	18.78±0.95^{ab}	11.9±1.96^{d}	8.87±1.71^{e}	1.54±0.60^{f}	14.80±1.39^{cd}	21.73±3.66^{a}	21.80±1.34^{a}	19.40±1.35^{a}
17	(Z)-2-penten-1-ol	0.00±0.00^{g}	0.62±0.15^{a}	0.37±0.06^{cd}	0.50±0.04^{abc}	0.16±0.06^{f}	0.23±0.02^{ef}	0.39±0.05^{cd}	0.36±0.05^{de}	0.28±0.09^{def}	0.40±0.09^{bcd}	0.53±0.06^{ab}
18	1-octen-3-ol	0.00±0.00^{f}	5.51±0.53^{ab}	4.48±0.76^{bcd}	5.82±0.21^{a}	2.31±1.26^{e}	3.68±0.76^{cd}	0.00±0.00^{f}	5.24±0.27^{ab}	1.86±0.95^{e}	3.43±0.63^{d}	4.82±0.38^{abc}
19	2-ethyl-1-hexanol	8.09±2.17^{a}	0.00±0.00^{d}	0.00±0.00^{d}	0.00±0.00^{d}	2.91±0.52^{c}	0.00±0.00^{d}	4.28±0.73^{b}	0.00±0.00^{d}	0.00±0.00^{d}	0.00±0.00^{d}	0.00±0.00^{d}
20	2-propyl-1-heptanol	2.04±0.55^{a}	0.53±0.04^{b}	0.45±0.04^{b}	0.43±0.06^{b}	0.00±0.00^{c}	0.00±0.00^{c}	0.34±0.07^{b}	0.00±0.00^{c}	0.00±0.00^{c}	0.00±0.00^{c}	0.00±0.00^{c}
21	3-methyl-1,5-pentanediol	0.00±0.00^{b}	0.08±0.01^{a}	0.00±0.00^{b}	0.00±0.00^{b}	0.00±0.00^{b}	0.00±0.00^{b}	0.00±0.00^{b}	0.00±0.00^{b}	0.00±0.00^{b}	0.00±0.00^{b}	0.00±0.00^{b}
	Aldehydes											

	Total content	0	19.95	26.63	19.97	33.33	35.08	0.87	2.63	20.57	19.98	16.5
22	Pentanal	0.00±0.00[e]	11.75±0.86[d]	18.85±1.96[c]	12.70±3.18[d]	27.96±3.79[b]	31.78±2.85[a]	0.00±0.00[e]	0.72±0.31[f]	10.62±0.23[de]	10.01±0.89[de]	7.53±2.25[e]
23	2-butenal	0.00±0.00[b]	0.58±0.05[a]	0.00±0.00[b]	0.00±0.00[b]	0.00±0.00[b]	0.00±0.00[b]	0.00±0.00[b]	0.00±0.00[b]	0.00±0.00[b]	0.00±0.00[b]	0.00±0.00[b]
24	Hexanal	0.00±0.00[d]	1.70±0.20[b]	1.34±0.14[bc]	0.36±0.14[cd]	0.52±0.11[cd]	0.00±0.00[d]	0.00±0.00[d]	0.00±0.00[d]	1.72±1.59[b]	2.83±0.42[a]	1.86±1.03[ab]
25	(E)-2-pentenal	0.00±0.00[c]	0.18±0.01[a]	0.00±0.00[c]	0.00±0.00[c]	0.00±0.00[c]	0.07±0.01[b]	0.00±0.00[c]	0.03±0.01[bc]	0.18±0.01[a]	0.23±0.05[a]	0.21±0.07[a]
26	Heptanal	0.00±0.00[d]	0.38±0.25c	0.00±0.00[d]	0.00±0.00[d]	0.00±0.00[d]	0.00±0.00[d]	0.00±0.00[d]	0.00±0.00[d]	1.75±0.48[a]	0.00±0.00[d]	0.77±0.05[b]
27	Octanal	0.00±0.00[d]	1.04±0.41[c]	1.61±0.41[abc]	0.97±0.19[c]	1.63±0.01[abc]	1.07±0.39[bc]	0.00±0.00[d]	0.00±0.00[d]	2.10±0.94[a]	2.07±0.11[a]	1.73±0.22[ab]
28	(E)-2-heptenal	0.00±0.00[e]	1.81±0.20[b]	1.26±0.27[cd]	1.58±0.11[bc]	0.86±0.20[d]	1.33±0.35[c]	0.00±0.00[e]	0.00±0.00[e]	0.88±0.34[d]	1.56±0.47[bc]	2.34±0.26[a]
29	Benzaldehyde	0.00±0.00[f]	1.80±0.67[bc]	2.22±0.89[ab]	2.75±0.61[a]	1.08±0.33[cde]	0.83±0.07[de]	0.47±0.05[ef]	0.61±0.07[ef]	1.05±0.22[cde]	1.18±0.29[cde]	1.45±0.14[cd]
30	Benzeneacetaldehyde	0.00±0.00[g]	0.42±0.05[a]	0.27±0.04[cd]	0.17±0.01[ef]	0.00±0.00[g]	0.00±0.00[g]	0.11±0.05[f]	0.32±0.03[bc]	0.35±0.11[b]	0.21±0.01[de]	0.29±0.02[bc]
31	(E)-2-hexenal	0.00±0.00[d]	0.29±0.09[bc]	0.36±0.10[b]	0.27±0.04[c]	0.00±0.00[d]	0.00±0.00[d]	0.29±0.02[bc]	0.95±0.02[a]	0.25±0.05[c]	0.32±0.05[bc]	0.32±0.03[bc]
32	Decanal	0.00±0.00[f]	0.00±0.00[f]	0.72±0.04[e]	1.17±0.11[d]	1.28±0.03[c]	0.00±0.00[f]	0.00±0.00[f]	0.00±0.00[f]	1.67±0.05[a]	1.57±0.11[b]	0.00±0.00[f]
	Ketones											
	Total content	2.16	2.75	2.83	3.2	1.51	3.37	0.83	2.12	1.31	2.35	3.16
33	3-pentanone	0.52±0.30[a]	0.00±0.00[b]	0.00±0.00[b]	0.00±0.00[b]	0.00±0.00[b]	0.00±0.00[b]	0.00±0.00[b]	0.00±0.00[b]	0.00±0.00[b]	0.00±0.00[b]	0.00±0.00[b]
34	2-heptanone	0.00±0.00[d]	0.00±0.00[d]	0.00±0.00[d]	0.62±0.08[a]	0.00±0.00[d]	0.43±0.05[b]	0.00±0.00[d]	0.30±0.07[c]	0.00±0.00[d]	0.00±0.00[d]	0.37±0.06[b]
35	1-octen-3-one	0.00±0.00[c]	0.15±0.01[b]	0.00±0.00[c]	0.00±0.00[c]	0.00±0.00[c]	0.18±0.02[a]	0.00±0.00[c]	0.00±0.00[c]	0.00±0.00[c]	0.00±0.00[c]	0.00±0.00[c]

36	6-methyl-5-hepten-2-one	0.00±0.00[e]	1.69±0.18[ab]	1.43±0.24[bc]	1.43±0.19[bc]	0.94±0.42[d]	1.43±0.21[bc]	0.00±0.00[e]	1.16±0.09[cd]	0.87±0.43[d]	1.80±0.18[ab]	2.06±0.18[a]
37	3-octen-2-one	0.00±0.00[c]	0.91±0.19[a]	1.12±0.30[a]	0.91±0.33[a]	0.28±0.25[bc]	0.96±0.16[a]	0.00±0.00[c]	0.42±0.23[b]	0.1±0.01[bc]	0.19±0.08[bc]	0.40±0.13[b]
38	2,3-octanedione	0.00±0.00[e]	0.00±0.00[e]	0.28±0.04[cd]	0.24±0.07[d]	0.29±0.02[bcd]	0.37±0.05[a]	0.00±0.00[e]	0.24±0.03[d]	0.34±0.04[ab]	0.36±0.01[a]	0.33±0.01[abc]
39	Acetophenone	1.64±0.10[a]	0.00±0.00[c]	0.00±0.00[c]	0.00±0.00[c]	0.00±0.00[c]	0.00±0.00[c]	0.83±0.24[b]	0.00±0.00[c]	0.00±0.00[c]	0.00±0.00[c]	0.00±0.00[c]
	Esters											
	Total content	0	0	0.07	0.06	0.20	0	0	0	0.07	0.07	0.21
40	Acetic acid, 2-ethylhexyl ester	0.00±0.00[b]	0.00±0.00[b]	0.00±0.00[b]	0.00±0.00[b]	0.16±0.08[a]	0.00±0.00[b]	0.00±0.00[b]	0.00±0.00[b]	0.00±0.00[b]	0.00±0.00[b]	0.17±0.02[a]
41	Acetic acid, pentyl ester	0.00±0.00[c]	0.00±0.00[c]	0.07±0.04[a]	0.06±0.01[ab]	0.04±0.01[ab]	0.00±0.00[c]	0.00±0.00[c]	0.00±0.00[c]	0.07±0.04[a]	0.07±0.02[a]	0.04±0.01[ab]
	Sulfides											
	Total content	4.86	1.68	0.79	0.61	0.72	0.45	15.27	30.51	0.28	0.22	1.08
42	Methyl thiocyanate	0.00±0.00[b]	0.00±0.00[b]	0.00±0.00[b]	0.00±0.00[b]	0.00±0.00[b]	0.00±0.00[b]	0.00±0.00[b]	0.44±0.08[a]	0.00±0.00[b]	0.00±0.00[b]	0.00±0.00[b]
43	2-methylbutyl isothiocyanate	0.00±0.00[b]	0.61±0.28[b]	0.34±0.11[b]	0.27±0.01[b]	0.32±0.02[b]	0.19±0.03[b]	0.74±0.13[b]	5.13±1.22[a]	0.00±0.00[b]	0.00±0.00[b]	0.33±0.05[b]
44	N-pentyl isothiocyanate	0.00±0.00[b]	0.00±0.00[b]	0.00±0.00[b]	0.00±0.00[b]	0.00±0.00[b]	0.00±0.00[b]	0.00±0.00[b]	0.38±0.18[a]	0.00±0.00[b]	0.00±0.00[b]	0.00±0.00[b]

45	Dimethyl disulfide	4.86±0.10[c]	0.00±0.00[d]	0.00±0.00[d]	0.00±0.00[d]	0.00±0.00[d]	0.00±0.00[d]	11.73±2.07[a]	7.21±0.49[b]	0.00±0.00[d]	0.00±0.00[d]	0.00±0.00[d]
46	Dimethyl trisulfide	0.00±0.00[c]	0.00±0.00[c]	0.00±0.00[c]	0.00±0.00[c]	0.00±0.00[c]	0.00±0.00[c]	2.33±0.61[b]	11.22±0.12[a]	0.00±0.00[c]	0.00±0.00[c]	0.00±0.00[c]
47	Allyl isothiocyanate	0.00±0.00[b]	0.37±0.20[b]	0.12±0.06[b]	0.12±0.06[b]	0.07±0.03[b]	0.05±0.01[b]	0.21±0.03[b]	3.83±1.32[a]	0.00±0.00[b]	0.00±0.00[b]	0.28±0.12[b]
48	Isobutyl isothiocyanate	0.00±0.00[c]	0.00±0.00[c]	0.00±0.00[c]	0.00±0.00[c]	0.00±0.00[c]	0.00±0.00[c]	0.26±0.62[b]	1.38±0.40[a]	0.00±0.00[c]	0.00±0.00[c]	0.00±0.00[c]
49	4-isothiocyanao -1-butene	0.00±0.00[e]	0.70±0.38[b]	0.33±0.14[cd]	0.22±0.07[de]	0.33±0.04[cd]	0.21±0.01[de]	0.00±0.00[e]	0.92±0.06[a]	0.28±0.04[cd]	0.22±0.04[de]	0.47±0.03[c]
	Acids											
	Total content	0	0.5	0.78	0	0	0.3	0	0.4	0	0.37	0.43
50	Diethyl-acetic acid	0.00±0.00[e]	0.5±0.11[b]	0.78±0.02[a]	0.00±0.00[e]	0.00±0.00[e]	0.30±0.16[d]	0.00±0.00[e]	0.40±0.02[bcd]	0.00±0.00[e]	0.37±0.02[cd]	0.43±0.03[bc]

Table S3. Relative odor activity values (ROAV ≥ 1) of broccoli volatile flavor compounds detected under different blanching methods and time.

Volatile compounds	Threshold (μg/kg)	Fragrance type	Un-blanched	Hot water blanching for 30 s	Hot water blanching for 60 s	Hot water blanching for 90 s	Hot water blanching for 120 s	Hot water blanching for 150 s	Steam blanching for 30 s	Steam blanching for 60 s	Steam blanching for 90 s	Steam blanching for 120 s	Steam blanching for 150 s
3-methyl-1-butanol	4	Whisky, malt, roasted aroma	9.79	—	—	—	—	—	1.15	—	—	—	—
1-hexanol	5.7	Resin, flower	5.04	1.05	0.93	1.39	0.32	1.15	2.9	0.09	0.28	0.36	2.62
Hexanal	5	Grass, butter, fat	—	6.17	3.72	0.62	0.81	—	—	—	2.06	3.61	7.72
Heptanal	3	Fat, citrus, rancidity	—	2.3	—	—	—	—	—	—	3.49	—	5.33
Octanal	0.7	Fat, soap, lemon	—	26.96	31.94	11.84	18.19	38.48	—	—	17.96	18.84	51.27
1-octen-3-one	4	Mushrooms, metals	—	0.68	—	—	—	1.13	—	—	—	—	—
3-methyl-1,5-pentanediol	0.3	Slightly greasy or waxy aroma	—	4.84	—	—	—	—	—	—	—	—	—
1-octen-3-ol	1	mushroom	—	100	62.22	49.74	18.05	92.64	—	4.67	11.14	21.85	100
Naphthalene	0.44	tar	—	4.95	—	—	—	—	—	—	1.36	—	—
Dimethyl disulfide	1.1	Onion, Chinese cabbage, rot	100	—	—	—	—	—	45.77	5.84	—	—	—
Dimethyl trisulfide	0.1	Sulfur, fish, Chinese cabbage	—	—	—	—	—	—	100	100	—	—	—
Decanal	0.1	Soap, orange peel, butter	—	—	100	100	100	—	—	—	100	100	—
Pentanal	8	Almond, malt, spicy	—	26.66	32.73	13.57	27.3	100	—	0.08	7.95	7.97	19.53
1-pentanol	150.2	Bread fragrance, wine fragrance, fruit fragrance	0.05	1.94	1.38	1.07	0.62	1.49	0.04	0.09	16.26	0.92	2.68

Note: — indicates that the compound was not detected.

Table S4. VIP values, ANOVA significance and relative contents of volatile compounds in broccoli under different blanching treatments

IDs	Volatile compounds	VIP values	*P*-value (ANOVA)	Relative content (Mean ± SD)/%										
				Un-blanched	Hot water blanching for 30 s	Hot water blanching for 60 s	Hot water blanching for 90 s	Hot water blanching for 120 s	Hot water blanching for 150 s	Steam blanching for 30 s	Steam blanching for 60 s	Steam blanching for 90 s	Steam blanching for 120 s	Steam blanching for 150 s
22	Pentanal	2.78	$P<0.001$	0.00±0.00^{e}	11.75±0.86^{d}	18.85±1.96^{c}	12.70±3.18^{d}	27.96±3.79^{b}	31.78±2.85^{a}	0.00±0.00^{e}	0.72±0.31^{f}	10.62±0.23^{de}	10.01±0.89^{de}	7.53±2.25^{e}
16	1-pentanol	2.33	$P<0.001$	0.36±0.12^{f}	16.02±1.47^{bc}	14.95±2.03^{cd}	18.78±0.95^{ab}	11.9±1.96^{d}	8.87±1.71^{e}	1.54±0.60^{f}	14.80±1.39^{cd}	21.73±3.66^{a}	21.80±1.34^{a}	19.40±1.35^{a}
45	Dimethyl disulfide	1.77	$P<0.001$	4.86±0.10^{c}	0.00±0.00^{d}	0.00±0.00^{d}	0.00±0.00^{d}	0.00±0.00^{d}	0.00±0.00^{d}	11.73±2.07^{a}	7.21±0.49^{b}	0.00±0.00^{d}	0.00±0.00^{d}	0.00±0.00^{d}
46	Dimethyl trisulfide	1.65	$P<0.001$	0.00±0.00^{c}	0.00±0.00^{c}	0.00±0.00^{c}	0.00±0.00^{c}	0.00±0.00^{c}	0.00±0.00^{c}	2.33±0.61^{b}	11.22±0.12^{a}	0.00±0.00^{c}	0.00±0.00^{c}	0.00±0.00^{c}
19	2-ethyl-1-hexanol	1.64	$P<0.001$	8.09±2.17^{a}	0.00±0.00^{d}	0.00±0.00^{d}	0.00±0.00^{d}	2.91±0.52^{c}	0.00±0.00^{d}	4.28±0.73^{b}	0.00±0.00^{d}	0.00±0.00^{d}	0.00±0.00^{d}	0.00±0.00^{d}
10	Pentadecane	1.62	$P<0.001$	0.00±0.00^{d}	0.31±0.05^{d}	0.00±0.00^{d}	0.00±0.00^{d}	0.00±0.00^{d}	0.00±0.00^{d}	2.25±0.37^{c}	0.00±0.00^{d}	3.88±0.28^{b}	4.90±0.64^{a}	0.00±0.00^{d}
18	1-octen-3-ol	1.44	$P<0.001$	0.00±0.00^{f}	5.51±0.53^{ab}	4.48±0.76^{bcd}	5.82±0.21^{a}	2.31±1.26^{e}	3.68±0.76^{cd}	0.00±0.00^{f}	5.24±0.27^{ab}	1.86±0.95^{e}	3.43±0.63^{d}	4.82±0.38^{abc}
26	Heptanal	1.25	$P<0.001$	0.00±0.00^{d}	0.38±0.25c	0.00±0.00^{d}	0.00±0.00^{d}	0.00±0.00^{d}	0.00±0.00^{d}	0.00±0.00^{d}	0.00±0.00^{d}	1.75±0.48^{a}	0.00±0.00^{d}	0.77±0.05^{b}
24	Hexanal	1.24	$P<0.001$	0.00±0.00^{d}	1.70±0.20^{b}	1.34±0.14^{bc}	0.36±0.14^{cd}	0.52±0.11^{cd}	0.00±0.00^{d}	0.00±0.00^{d}	0.00±0.00^{d}	1.72±1.59^{b}	2.83±0.42^{a}	1.86±1.03^{ab}
7	2,6,10-	1.18	$P<0.001$	0.00±0.00^{d}	0.31±0.12^{cd}	0.69±0.10^{c}	0.71±0.06^{c}	1.59±0.35^{b}	2.28±0.50^{a}	0.44±0.05^{cd}	0.48±0.17^{cd}	1.72±0.72^{ab}	2.03±0.20^{ab}	1.81±0.14^{ab}

	trimethyl tridecane													
43	2-methylbutyl isothiocyanate	1.12	*P*＜0.001	0.00±0.00^{b}	0.61±0.28^{b}	0.34±0.11^{b}	0.27±0.01^{b}	0.32±0.02^{b}	0.19±0.03^{b}	0.74±0.13^{b}	5.13±1.22^{a}	0.00±0.00^{b}	0.00±0.00^{b}	0.33±0.05^{b}
12	1-hexanol	1.11	*P*＜0.001	1.27±1.11^{b}	0.33±0.05^{c}	0.38±0.03^{c}	0.93±0.19^{bc}	0.23±0.03^{c}	0.26±0.04^{c}	3.85±0.28^{a}	0.55±0.10^{c}	0.27±0.02^{c}	0.32±0.03^{c}	0.72±0.38^{bc}
29	Benzaldehyde	1.11	*P*＜0.001	0.00±0.00^{f}	1.80±0.67^{bc}	2.22±0.89^{ab}	2.75±0.61^{a}	1.08±0.33^{cde}	0.83±0.07^{de}	0.47±0.05^{ef}	0.61±0.07^{ef}	1.05±0.22^{cde}	1.18±0.29^{cde}	1.45±0.14^{cd}
13	(Z)-3-hexen-1-ol	1.08	*P*＜0.001	3.33±0.39^{a}	0.00±0.00^{d}	0.00±0.00^{d}	0.00±0.00^{d}	0.00±0.00^{d}	0.00±0.00^{d}	0.74±0.14^{c}	1.90±0.29^{b}	0.00±0.00^{d}	0.00±0.00^{d}	0.00±0.00^{d}
32	Decanal	1.05	*P*＜0.001	0.00±0.00^{f}	0.00±0.00^{f}	0.72±0.04^{e}	1.17±0.11^{d}	1.28±0.03^{c}	0.00±0.00^{f}	0.00±0.00^{f}	0.00±0.00^{f}	1.67±0.05^{a}	1.57±0.11^{b}	0.00±0.00^{f}
47	Allyl isothiocyanate	0.97	*P*＜0.001	0.00±0.00^{b}	0.37±0.20^{b}	0.12±0.06^{b}	0.12±0.06^{b}	0.07±0.03^{b}	0.05±0.01^{b}	0.21±0.03^{b}	3.83±1.32^{a}	0.00±0.00^{b}	0.00±0.00^{b}	0.28±0.12^{b}
28	(E)-2-heptenal	0.97	*P*＜0.001	0.00±0.00^{e}	1.81±0.20^{b}	1.26±0.27^{cd}	1.58±0.11^{bc}	0.86±0.20^{d}	1.33±0.35^{c}	0.00±0.00^{e}	0.00±0.00^{e}	0.88±0.34^{d}	1.56±0.47^{bc}	2.34±0.26^{a}
27	Octanal	0.93	*P*＜0.001	0.00±0.00^{d}	1.04±0.41^{c}	1.61±0.41^{abc}	0.97±0.19^{c}	1.63±0.01^{abc}	1.07±0.39^{bc}	0.00±0.00^{d}	0.00±0.00^{d}	2.10±0.94^{a}	2.07±0.11^{a}	1.73±0.22^{ab}
50	Diethyl-acetic acid	0.91	*P*＜0.001	0.00±0.00^{e}	0.5±0.11^{b}	0.78±0.02^{a}	0.00±0.00^{e}	0.00±0.00^{e}	0.30±0.16^{d}	0.00±0.00^{e}	0.40±0.02^{bcd}	0.00±0.00^{e}	0.37±0.02^{cd}	0.43±0.03^{bc}
36	6-methyl-5-hepten-2-one	0.87	*P*＜0.001	0.00±0.00^{e}	1.69±0.18^{ab}	1.43±0.24^{bc}	1.43±0.19^{bc}	0.94±0.42^{d}	1.43±0.21^{bc}	0.00±0.00^{e}	1.16±0.09^{cd}	0.87±0.43^{d}	1.80±0.18^{ab}	2.06±0.18^{a}
20	2-propyl-1-hept	0.83	*P*＜0.001	2.04±0.55^{a}	0.53±0.04^{b}	0.45±0.04^{b}	0.43±0.06^{b}	0.00±0.00^{c}	0.00±0.00^{c}	0.34±0.07^{b}	0.00±0.00^{c}	0.00±0.00^{c}	0.00±0.00^{c}	0.00±0.00^{c}

	anol													
8	Hexadecane	0.81	$P<0.001$	0.00±0.00^{d}	0.00±0.00^{d}	0.51±0.02^{a}	0.42±0.09^{b}	0.00±0.00^{d}	0.35±0.03^{c}	0.00±0.00^{d}	0.32±0.01^{c}	0.00±0.00^{d}	0.00±0.00^{d}	0.53±0.01^{a}
34	2-heptanone	0.81	$P<0.001$	0.00±0.00^{d}	0.00±0.00^{d}	0.00±0.00^{d}	0.62±0.08^{a}	0.00±0.00^{d}	0.43±0.05^{b}	0.00±0.00^{d}	0.30±0.07^{c}	0.00±0.00^{d}	0.00±0.00^{d}	0.37±0.06^{b}
37	3-octen-2-one	0.80	$P<0.001$	0.00±0.00^{c}	0.91±0.19^{a}	1.12±0.30^{a}	0.91±0.33^{a}	0.28±0.25^{bc}	0.96±0.16^{a}	0.00±0.00^{c}	0.42±0.23^{b}	0.1±0.01^{bc}	0.19±0.08^{bc}	0.40±0.13^{b}
23	2-butenal	0.78	$P<0.001$	0.00±0.00^{b}	0.58±0.05^{a}	0.00±0.00^{b}	0.00±0.00^{b}	0.00±0.00^{b}	0.00±0.00^{b}	0.00±0.00^{b}	0.00±0.00^{b}	0.00±0.00^{b}	0.00±0.00^{b}	0.00±0.00^{b}
11	3-methyl-1-butanol	0.66	$P<0.001$	1.73±0.57^{a}	0.00±0.00^{c}	0.00±0.00^{c}	0.00±0.00^{c}	0.00±0.00^{c}	0.00±0.00^{c}	1.07±0.18^{b}	0.00±0.00^{c}	0.00±0.00^{c}	0.00±0.00^{c}	0.00±0.00^{c}
39	Acetophenone	0.64	$P<0.001$	1.64±0.10^{a}	0.00±0.00^{c}	0.00±0.00^{c}	0.00±0.00^{c}	0.00±0.00^{c}	0.00±0.00^{c}	0.83±0.24^{b}	0.00±0.00^{c}	0.00±0.00^{c}	0.00±0.00^{c}	0.00±0.00^{c}
49	4-isothiocyanao-1-butene	0.62	$P<0.001$	0.00±0.00^{e}	0.70±0.38^{b}	0.33±0.14^{cd}	0.22±0.07^{de}	0.33±0.04^{cd}	0.21±0.01^{de}	0.00±0.00^{e}	0.92±0.06^{a}	0.28±0.04^{cd}	0.22±0.04^{de}	0.47±0.03^{c}
14	1-octanol	0.62	$P<0.001$	0.00±0.00^{f}	1.10±0.23^{b}	0.87±0.15^{bcd}	0.83±0.19^{cd}	0.67±0.04^{de}	0.56±0.06^{e}	0.74±0.14^{de}	1.41±0.26^{a}	1.05±0.04^{bc}	0.90±0.02^{bcd}	1.06±0.09^{bc}
48	Isobutyl isothiocyanate	0.58	$P<0.001$	0.00±0.00^{c}	0.00±0.00^{c}	0.00±0.00^{c}	0.00±0.00^{c}	0.00±0.00^{c}	0.00±0.00^{c}	0.26±0.62^{b}	1.38±0.40^{a}	0.00±0.00^{c}	0.00±0.00^{c}	0.00±0.00^{c}
1	P-xylene	0.55	$P<0.001$	0.16±0.02^{c}	0.00±0.00^{d}	0.21±0.02^{b}	0.31±0.02^{a}	0.00±00^{d}	0.16±0.05^{c}	0.00±0.00^{d}	0.03±0.01^{d}	0.00±0.00^{d}	0.00±0.00^{d}	0.00±0.00^{d}
40	Acetic acid, 2-ethylhexyl ester	0.55	$P<0.001$	0.00±0.00^{b}	0.00±0.00^{b}	0.00±0.00^{b}	0.00±0.00^{b}	0.16±0.08^{a}	0.00±0.00^{b}	0.00±0.00^{b}	0.00±0.00^{b}	0.00±0.00^{b}	0.00±0.00^{b}	0.17±0.02^{a}
4	D-limonene	0.51	$P<0.001$	0.00±0.00^{c}	0.00±0.00^{c}	0.00±0.00^{c}	0.00±0.00^{c}	0.22±0.07^{a}	0.16±0.01^{b}	0.00±0.00^{c}	0.00±0.00^{c}	0.00±0.00^{c}	0.21±0.05^{ab}	0.17±0.01^{b}

38	2,3-octanedione	0.51	$P<0.001$	0.00±0.00^{e}	0.00±0.00^{e}	0.28±0.04^{cd}	0.24±0.07^{d}	0.29±0.02^{bcd}	0.37±0.05^{a}	0.00±0.00^{e}	0.24±0.03^{d}	0.34±0.04^{ab}	0.36±0.01^{a}	0.33±0.01^{abc}
31	(E)-2-hexenal	0.51	$P<0.001$	0.00±0.00^{d}	0.29±0.09^{bc}	0.36±0.10^{b}	0.27±0.04^{c}	0.00±0.00^{d}	0.00±0.00^{d}	0.29±0.02^{bc}	0.95±0.02^{a}	0.25±0.05^{c}	0.32±0.05^{bc}	0.32±0.03^{bc}
17	(Z)-2-penten-1-ol	0.50	$P<0.001$	0.00±0.00^{g}	0.62±0.15^{a}	0.37±0.06^{cd}	0.50±0.04^{abc}	0.16±0.06^{f}	0.23±0.02^{ef}	0.39±0.05^{cd}	0.36±0.05^{de}	0.28±0.09^{def}	0.40±0.09^{bcd}	0.53±0.06^{ab}
30	Benzeneacetaldehyde	0.50	$P<0.001$	0.00±0.00^{g}	0.42±0.05^{a}	0.27±0.04^{cd}	0.17±0.01^{ef}	0.00±0.00^{g}	0.00±0.00^{g}	0.11±0.05^{f}	0.32±0.03^{bc}	0.35±0.11^{b}	0.21±0.01^{de}	0.29±0.02^{bc}
35	1-octen-3-one	0.45	$P<0.001$	0.00±0.00^{c}	0.15±0.01^{b}	0.00±0.00^{c}	0.00±0.00^{c}	0.00±0.00^{c}	0.18±0.02^{a}	0.00±0.00^{c}	0.00±0.00^{c}	0.00±0.00^{c}	0.00±0.00^{c}	0.00±0.00^{c}
33	3-pentanone	0.44	$P<0.001$	0.52±0.30^{a}	0.00±0.00^{b}	0.00±0.00^{b}	0.00±0.00^{b}	0.00±0.00^{b}	0.00±0.00^{b}	0.00±0.00^{b}	0.00±0.00^{b}	0.00±0.00^{b}	0.00±0.00^{b}	0.00±0.00^{b}
3	O-xylene	0.43	$P<0.001$	0.30±0.15^{a}	0.12±0.04^{cde}	0.22±0.05^{abc}	0.00±0.00^{f}	0.22±0.07^{ab}	0.21±0.11^{abcd}	0.00±0.00^{f}	0.11±0.03^{de}	0.00±0.00^{f}	0.09±0.01^{ef}	0.14±0.02^{bcde}
15	1-penten-3-ol	0.42	$P<0.001$	0.47±0.26^{b}	0.37±0.06^{bcd}	0.27±0.05^{bcde}	0.4±0.08^{bc}	0.17±0.07^{de}	0.22±0.06^{cde}	0.70±0.15^{a}	0.31±0.14^{bcde}	0.15±0.03^{f}	0.23±0.04^{cde}	0.29±0.03^{bcde}
9	Tetradecane	0.40	$P<0.001$	0.82±0.21^{a}	0.20±0.12^{c}	0.20±0.01^{c}	0.22±0.09^{c}	0.34±0.02^{bc}	0.25±0.02^{c}	0.73±0.16^{a}	0.36±0.03^{bc}	0.48±0.13^{b}	0.33±0.03^{bc}	0.31±0.01^{bc}
6	Naphthalene	0.40	$P<0.001$	0.00±0.00^{c}	0.12±0.02^{a}	0.00±0.00^{c}	0.00±0.00^{c}	0.00±0.00^{c}	0.00±0.00^{c}	0.00±0.00^{c}	0.00±0.00^{c}	0.10±0.03b	0.00±0.00^{c}	0.00±0.00^{c}
25	(E)-2-pentenal	0.39	$P<0.001$	0.00±0.00^{c}	0.18±0.01^{a}	0.00±0.00^{c}	0.00±0.00^{c}	0.00±0.00^{c}	0.07±0.01^{b}	0.00±0.00^{c}	0.03±0.01^{bc}	0.18±0.01^{a}	0.23±0.05^{a}	0.21±0.07^{a}
42	Methyl thiocyanate	0.34	$P<0.001$	0.00±0.00^{b}	0.00±0.00^{b}	0.00±0.00^{b}	0.00±0.00^{b}	0.00±0.00^{b}	0.00±0.00^{b}	0.00±0.00^{b}	0.44±0.08^{a}	0.00±0.00^{b}	0.00±0.00^{b}	0.00±0.00^{b}
44	N-pentyl isothiocyanate	0.31	$P<0.001$	0.00±0.00^{b}	0.00±0.00^{b}	0.00±0.00^{b}	0.00±0.00^{b}	0.00±0.00^{b}	0.00±0.00^{b}	0.00±0.00^{b}	0.38±0.18^{a}	0.00±0.00^{b}	0.00±0.00^{b}	0.00±0.00^{b}
5	P-cymene	0.28	$P<0.001$	0.00±0.00^{b}	0.07±0.04^{a}	0.00±0.00^{b}	0.00±0.00^{b}	0.09±0.06^{a}	0.06±0.02^{a}	0.00±0.00^{b}	0.00±0.00^{b}	0.00±0.00^{b}	0.00±0.00^{b}	0.00±0.00^{b}

21	3-methyl-1,5-pentanediol	0.28	$P < 0.001$	0.00±0.00[b]	0.08±0.01[a]	0.00±0.00[b]	0.00±0.00[b]	0.00±0.00[b]	0.00±0.00[b]	0.00±0.00[b]	0.00±0.00[b]	0.00±0.00[b]	0.00±0.00[b]	0.00±0.00[b]
41	Acetic acid, pentyl ester	0.23	$P < 0.001$	0.00±0.00[c]	0.00±0.00[c]	0.07±0.04[a]	0.06±0.01[ab]	0.04±0.01[ab]	0.00±0.00[c]	0.00±0.00[c]	0.00±0.00[c]	0.07±0.04[a]	0.07±0.02[a]	0.04±0.01[ab]
2	Nonane	0.20	$P < 0.001$	0.00±0.00[c]	0.00±0.00[c]	0.00±0.00[c]	0.03±0.01[b]	0.00±0.00[c]	0.00±0.00[c]	0.00±0.00[c]	0.00±0.00[c]	0.00±0.00[c]	0.05±0.04[a]	0.00±0.00[c]

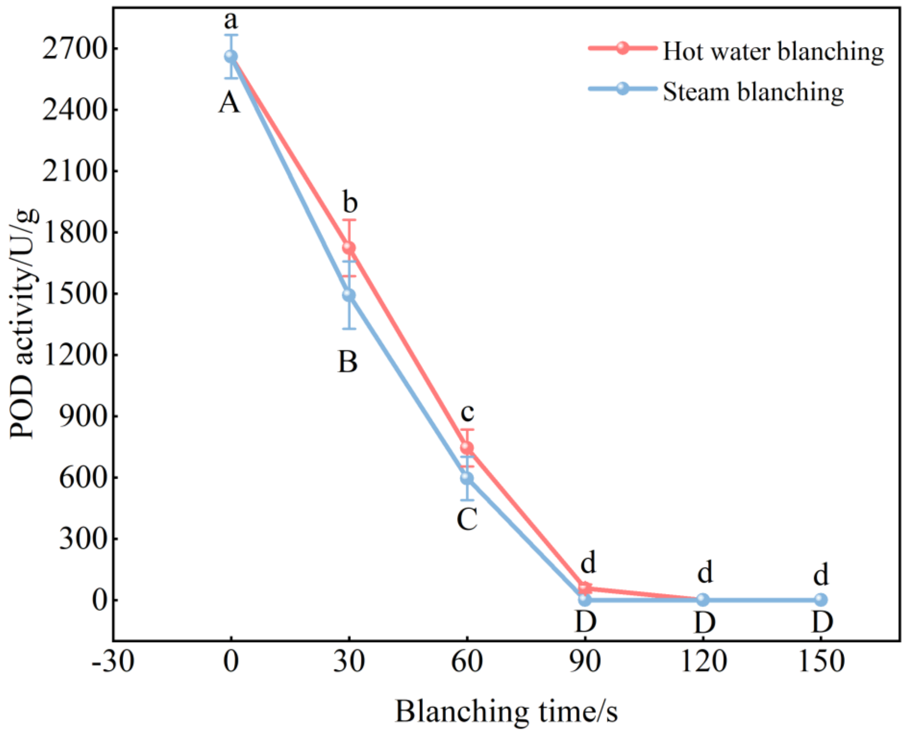

Figure S1. Peroxidase activity of broccoli under different blanching times.

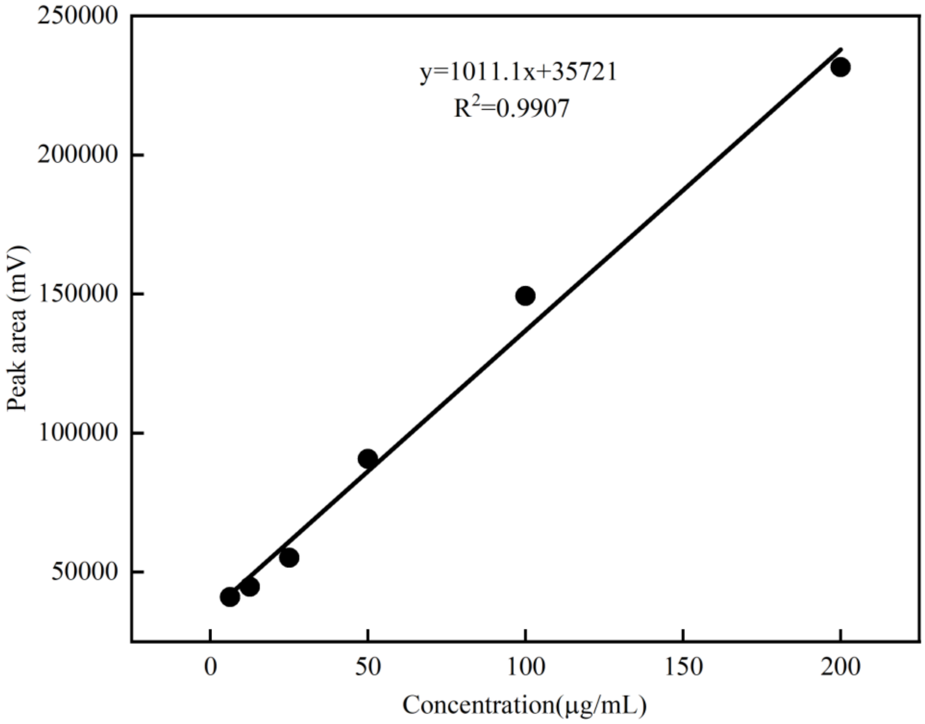

Figure S2. The standard curve of sulforaphane by HPLC analysis.
